# BONE GEOMETRY AND PHYSICAL ACTIVITY IN CHILDREN AND ADOLESCENTS: SYSTEMATIC REVIEW

**DOI:** 10.1590/1984-0462/;2018;36;2;00005

**Published:** 2018-01-15

**Authors:** Tathyane Krahenbühl, Roseane de Fátima Guimarães, Antonio de Azevedo Barros, Ezequiel Moreira Gonçalves

**Affiliations:** a Universidade Federal de Goiás, Goiânia, GO, Brasil.; b Universidade Estadual de Campinas, Campinas, SP, Brasil.

**Keywords:** Exercise, Sports, Bone density, Adolescents, Athletes, Exercício, Esportes, Densidade óssea, Adolescentes, Atletas

## Abstract

**Objective:**

To perform a systematic review on the practice of physical activity and/or sports in health and its influence on bone geometry of healthy children and adolescents.

**Data source:**

The method used as reference was the Preferred Reporting Items for Systematic Reviews and Meta-Analyses (PRISMA). Databases searched for articles published from 2006 to 2016, with “Bone geometry” AND (Sport* OR Exercise* OR “Physical Activity”) as descriptors, were PubMed, BIREME/LILACS and SciELO.

**Data syntheses:**

After the selection, 21 articles were included. Most studies stated that practice of physical activity and/or sports was beneficial for bone geometry and bone mineral density. Only two studies presented values of bone parameters for control individuals better than those of swimmers. Physical activities and sports studied were: gymnastics (n=7), rhythmic gymnastics (n=2), tennis (n=1), soccer (n=3), *capoeira* (n=1), swimming (n=4), cycling (n=0), jumping activities (n=2), studies relating physical activity with isokinetic peak torque (n=1), physical activity measured by questionnaire (n=4), and additional physical education classes (n=2).

**Conclusions:**

Among the sports and physical activities found, gymnastics, soccer, and more intense physical activity assessed by questionnaires were mentioned along with better results in bone geometry compared to the absence of physical activity, whereas swimming and jumping exercises did not influence it. Therefore, sports activities with weight bearing and those practiced more frequently and intensively are beneficial for bone geometry.

## RESUMO

**Objetivo:**

Verificar a influência da prática de atividade física e/ou esportes na geometria óssea de crianças e adolescentes saudáveis.

**Fonte de dados:**

Foi realizada uma revisão sistemática, utilizando como referência o método *Preferred Reporting Items for Systematic Reviews and Meta-Analyses* (PRISMA). Foram utilizadas as bases de buscas PubMed, Biblioteca Regional de Medicina/Literatura Latino-Americana e do Caribe em Ciências da Saúde (BIREME/LILACS) e *Scientific Electronic Library Online* (SciELO), para levantamento de artigos publicados de 2006 a 2016, e os seguintes descritores: “Bone geometry” AND (Sport* OR Exercise* OR “Physical Activity”).

**Síntese dos dados:**

Após a seleção, foram incluídos 21 artigos. A maioria dos estudos demonstrou que a prática de atividade física e/ou esportes foi benéfica do ponto de vista da geometria e densidade mineral óssea; apenas dois estudos apresentaram valores dos parâmetros ósseos dos indivíduos controles melhores do que os praticantes de natação. As atividades físicas e esportes encontrados foram: ginástica artística (n=7), ginástica rítmica (n=2), tênis (n=1), futebol (n=3), capoeira (n=1), natação (n=4), ciclismo (n=1), atividades com saltos (n=2), estudos relacionando atividade física com pico de torque isocinético (n=1), atividade física em geral, tempo presente ou passado, mensurado por questionário (n=4) e aulas adicionais de educação física (n=2).

**Conclusões:**

Dentre os esportes e atividades físicas encontradas, a ginástica, o futebol e a prática de atividade física mais intensa avaliada por questionário resultaram em geometria óssea melhor em comparação à não prática de atividade física, enquanto que a natação e exercícios de saltos não influenciaram a geometria óssea. Portanto, atividades esportivas com sobrecarga corporal, avaliadas como mais intensas e mais frequentes, exercem efeito benéfico sobre a geometria óssea.

## INTRODUCTION

Bone tissue goes through countless changes in childhood and adolescence, and such stages are characterized by intense physical growth and overall body development. These changes occur mainly because of the linear increase in bone tissue happening in such periods, which reflects the predominance of bone deposition to the detriment of bone resorption.^1^ ^,^ ^2^ ^,^ ^3^ Bone structural integrity depends on parameters such as total bone mass, properties of constituent tissue, and bone geometry.^4^

Bone geometry is defined by bone tissue parameters such as bone diameter, bone cross-sectional area and total bone area, and by bone architecture indicators such as cross-sectional moment of inertia (CSMI), which is defined as the structural stiffness index reflecting the mass distribution around the core of a structural element, i.e., the sum of pixels mass at each point of the profile times the square of distance between profile mass core and intertrabecular connectivity.^5^ ^,^ ^6^ Material properties of bone are usually described by variables such as modulus of elasticity, which indicates bone material rigidity by its ability to withstand stress-an indicator of bone strength-, and the capacity of absorb energy, measured by bone volume unit. Therefore, bone geometry can be defined by where and how the material that makes up bone tissue structure is distributed.^7^ Factors such as intensity and orientation of bone modeling or even bone tissue removal, can alter bone geometry.^6^

In addition, genetics, hormonal status, sun exposure and diet may influence bone tissue constitution along with regular physical activity or sports practice, especially with body overload, which plays an important role in bone mass and strength development and maintenance. In addition to this, it is suggested that bone responsiveness to mechanical load increased depends on the bone resorption induced by growth; that is, physical activity during growth benefits the bone structure mineral accumulation process.^3^ ^,^ ^8^ ^,^ ^9^

Physical activity affects bone density and geometry because bone tissue self-organizes according to loads from specific physical-sport activities. However, the effects of different sports on bone health are not fully understood yet, as they may vary according to intensity of impact and type of activity - with (gymnastics, soccer, volleyball) or without body overload (swimming).^10^ ^,^ ^11^ ^,^ ^12^ ^,^ ^13^ Furthermore, there are indications that prepubescent and pubertal individuals who perform physical exercises with demands of body overload have geometrically larger and stronger bones.^14^

Most studies evaluating the effect of mechanical load on bone growth have focused on bone mineral density (BMD) and bone mineral content (BMC) parameters. However, recently, bone geometry parameters have been used to verify the bone quality in children and adolescents. There are several methods to assess bone geometry, some demonstrating a close relationship with bone quality, such as bone modeling intensity, removal of mechanically significant components that make up bone tissue, bone diameters and cross-sectional area, moment of inertia, and intertrabecular connectivity, among others.^6^ Which physical and sports activities interfere in bone geometry is a matter still to be resolved. Therefore, the objective of this study was to verify, through a systematic review, the influence of physical and/or sports activity on bone geometry in healthy children and adolescents.

## METHOD

This study is a systematic review of the literature addressing the influence of practice of physical activity and/or sports on the health and bone geometry of healthy children and adolescents. The method used as reference was the Preferred Reporting Items for Systematic Reviews and Meta-Analyzes (PRISMA).^15^ First, we searched PubMed, Regional Library of Medicine/Latin American and Caribbean Literature in Sciences (BIREME/LILACS), and Scientific Electronic Library Online (SciELO) databases for articles published from 2006 to 2016.

The search was carried out by two authors at different times, in English and Portuguese. The following descriptors, words and combinations for data search were used: “Bone geometry” AND (Sport* OR Exercise* OR “Physical Activity”). Inclusion criteria were:

- sample of individuals aging up to 18 (children and adolescents);
- sample of physical activity practitioners and/or athletes;
- only human beings;
- not bearing diseases;
- original articles; and
- articles aiming to verify the influence and/or effects of physical activity and/or sports on bone geometry.

All types of intervention with physical activity, exercise or sports were included in the sample, with no distinction of load, intensity or frequency; however, the articles that did not show results referring to physical activity compared to bone geometry were excluded.

**Figure 1:**
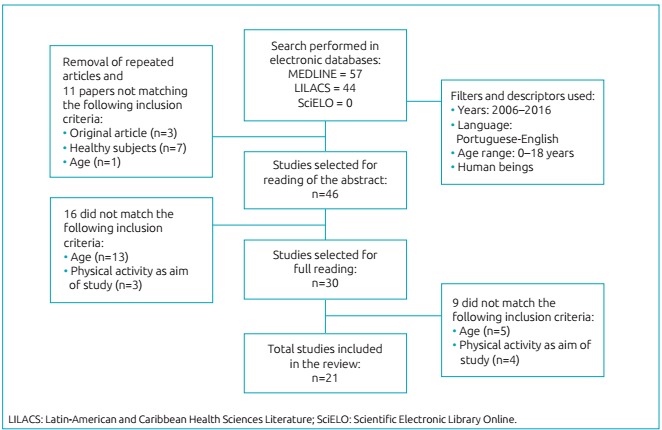
Flowchart showing the steps of process of selection of studies for the review.

During the search phases, authors also performed analysis of headings, consequent removal of duplicates and reading of abstracts. Therefore, the selection of complete articles for reading and, finally, to be included in the review was made according to what Figure 1 shows.

Important to emphasize, before discussing the results found, that three different methods of bone geometry evaluation are usually found. The method of peripheral quantitative computed tomography (pQCT) allows a three-dimensional evaluation of cortical and trabecular parts of the bone, thus allowing bone density, geometry and strength estimation, with accuracy for changes in body overload.^16^ Dual-energy X-ray absorptiometry (DXA), on the other hand, has often been used because it emits a lower dose of radiation and, unlike pQCT, cannot distinguish trabecular and cortical bones. In order to measure bone geometry, additional software is needed.^17^ Programs used are Hip Structural Analysis (HSA), an Hologic-model software, and Advanced Hip Assessment (AHA) for GE-Lunar machines.^18^ In general, bone geometry and density parameters provided by DXA are positively correlated with assessments by pQCT.^17^ Finally, bone geometry can be analyzed by Magnetic Resonance Imaging (MRI), which is commonly used to target the musculoskeletal system and pathologies related. A MRI protocol can be directly compared to pQCT density measurements, besides not using ionizing radiation and being a more sensitive method to tissue pathological changes.^19^

## RESULTS

Twenty-one articles matching the inclusion criteria were found, and the main focus of this study was to review the studies conducted with healthy children and adolescents and those who physical activities or sports. Among studies included, 13 had cross-sectional design (Table 1) and eight were longitudinal (Table 2).

**Table 1:** Cross-sectional studies included in the research, along with their samples’ characteristics, methods and results.

Study	Sample	Methods	Results
20	females (n=103) ±7.8 years (♀)	DXA, HSA	Low positive correlation of PA with femur bone area and vertical jumps. BMC of total leg, femur diameter and cross-sectional area positively correlated to PT.
21	Hgym (n=28), Lgym (n=28), Nogym (n=28) ± 7.9 years (♀)	DXA, pQCT	DXA: higher BMC for gymnasts. pQCT: gymnasts with higher BMC values, total bone density, strength and deformation index.
14	Low PA (n=41) Alta AF (n=25) ±10.0 years (♂♀)	DXA, pQCT	DXA: group with more PA and higher BMC values for radius, femur and whole body. pQCT: group with more PA involving bone cross-sectional area and circumference (white males).
22	RG (n=26), CON (n=23), ±10.5 years (♀)	pQCT	RG with higher total and cortical BMC, cortical area, bone and muscle deformation, thickness and circumference.
23	Children (n=424) 9-11 years (♂♀)	pQCT	PA related to total and cortical areas, bone density, deformation index (♂) and strength index (♂♀). VHJ related to bone strength index and cortical area (♀).
24	Low PA (n=25) Medium PA (n=17) High PA (n=18) ±11.0 years (♀)	pQCT	High PA and greater cortical thickness, cross-sectional area, bone deformation index, and total, cortical, volumetric BMC. Medium PA and higher cortical BMC and bone deformation index compared to low PA.
25	AG (n=28) Nogym (n=28) Tanner I e II (♀)	DXA	AG with higher BMD and BMC, periosteum width, density area, bone strength, thickness and diameter indexes.
26	Low, medium, and high PA (n=465) 8-13 years (♀)	pQCT	Longer duration, higher frequency and load of PA and high values of periosteal and endocortical circumference, bone strength and deformation index.
27	SW (n=41), FOOT (n=37), CYC (n=29), CON (n=14) 12-14 years (♂)	DXA,HSA	Athletes with higher BMD and BMC for all bones (except lumbar spine and arms).
28	AG (n=23), CON (n=23) ±13.3 years (♀)	DXA, HSA	AG with higher total BMD value for arms, legs, femur, lumbar spine, radius, cross-sectional area, modular session, and bone thickness.
29	AG (n=20), RG (n=20), NAT (n=20) CON (n=20) ±13.8 years (♀)	DXA, HSA	AG with higher BMD values for all bones compared to SW and CON. AG with higher BMD values for lumbar spine and radius compared to RG. RG with higher values for femur compared to SW and CON. AG and RG with lower values for BR compared to SW and CON.
30	SW (n=26), FOOT (n=32), CON (n=15) ±16.0 years (♀)	DXA, HSA	FOOT with higher density parameters compared to SW. FOOT higher bone strength and density parameters compared to SW and CON. SW presented low hip Z-score, below average.
31	Exgym (n=16), Nogym (n=13) ±16.2 years (♀)	pQCT	Ex-gymnasts with greater bone cross-sectional values, bone strength indexes, and volumetric density.

CON: control; Hgym: high-intensity gymnasts; Lgym: low-intensity gymnasts; Nogym: not gymnasts; Exgym: ex-gymnast; PA: physical activity; SW: swimmers; FOOT: football players; CYC: cyclists; AG: artistic gymnastic; RG: rhythmic gymnastics; DXA: Dual-energy X-ray absorptiometry; HSA: hip structural analysis; pQCT: peripheral quantitative computed tomography; MRI: magnetic resonance imaging; QUS: quantitative ultrasound; BMD: bone mineral density; BMC: bone mineral content; VHJ: vertical jumps; PT: isokinetic peak torque; BR: Buckling Ratio; ♀: females; ♂: males.

**Table 2:** Longitudinal studies included in the research, along with their samples’ characteristics, methods and results.

Study	Sample	Methods	Intervention	Results
32	CON (n=13), LJ (n=13), HJ (n=13) ±7.8 years (♀)	DXA, *analysis software* MRI	HJ=28 cm; LJ=14 cm; 10 series of 5 repetitions, 3x/week. T=8 months	No differences between groups.
9	Interv (n=42) e CON (n=43) ±7.9 years (♀)	DXA, HSA	200-min additional PE class per week, T=2 years.	No differences between groups.
8	Interv (n=72) e CON (n=55) ±7.9 years (♂)	DXA, HSA	200-min additional PE class per week, T=2 years.	Higher intervention compared to CON in BMD of third lumbar vertebra (cm).
33	Hgym (n=28), Lgym (n=28) e Nogym (n=28) ±7.9 years (♀)	DXA, pQCT	Hgym=6-16h/week Lgym=1-5h/week T=6 months.	DXA: gymnasts showing higher values for arm BMC. pQCT: gymnasts showing higher values for total cortical area and medullar area, bone strength and deformation index, cortical thickness, total bone density and content.
34	AG (n=28), Exgym (n=64), Nogym (n=73) 4-10 years (♂♀)	DXA, HSA	Recreational gymnastics ≥45min/week T=4 years	Gymnasts with higher values of cross-sectional area and modular section. Ex-gymnasts did not differ from CON.
35	Capoeira practitioners (n=104), CON (n=68) ±10.5 years (♂)	DXA, calcaneus QUS.	10 min/session, 3x/week *Capoeira* = movements, jumps, kicks, low kicks. T=9 months	*Capoeira* practitioners with jumping exercises had increased parameters for ultrasound, periosteum circumference/thickness radius in lumber spine compared to CON.
36	Tennis players (n=45) 10-17 years (♀)	MRI	Minimum 2h/week T=12 months	Values of most used arm in game compared to other arm in BMC, total area and bone cortical/cross-sectional muscle area.
37	SW (n=26), FOOT (n=32), CON (n=15) ±16.0 years (♀)	DXA, HSA	SW=260 sessions/year, 10h/week, 1500km (total of study) FOOT=225 sessions/year, 2h/day, 39 weeks T=8 months	FOOT increased total BMD, lumbar spine, hips and femur, whole body Z-score, and femur area, thickness, and strength index. SW had increased BMD in intertrochanteric and BR, decreased whole body and femur Z-score.

PE: physical education; Interv: intervention; CON: control; h/week: hours per week; T: time between evaluations; Exgym: ex-gymnast; Hgym: high-intensity gymnasts; Lgym: low-intensity gymnasts; Nogym: not gymnasts; SW: swimmers; FOOT: football players; LJ: low jumps; HJ: high jumps; DXA: Dual-energy X-ray absorptiometry; HSA: hip structural analysis; pQCT: peripheral quantitative computed tomography; MRI: magnetic resonance imaging; QUS: quantitative ultrasound; BMD: bone mineral density; BMC: bone mineral content; BR: Buckling Ratio; ♀: females; ♂: males.

Overall, 90% of the studies included (19 articles) stated significant differences between the active and the control group (not regular physical activity practitioners), which shows that the practice of physical activity and/or sports was beneficial from the point of view of bone geometry and BMD. However, two studies had values of bone parameters in control subjects better than those of active individuals related to girls who practice swimming, while two studies did not find differences between groups after a period of intervention.

Physical activity and sports were: gymnastics (n=7), rhythmic gymnastics (n=2), tennis (n=1), soccer/football (n=3), *capoeira* (n=1), swimming (n=4), cycling (n=1), activities with jumps (n=2), studies relating physical activity with isokinetic peak torque (n=1), physical activities in general in past or present time measured by questionnaire (n=4), and additional physical education classes (n=2). Among physical activities and sports found, gymnastics, soccer, capoeira, tennis, and physical activity in general (questionnaires) had better results on bone geometry than those observed in control groups. When it came to swimmers, results were inferior not only to other sports (soccer/football and gymnastics), but also to controls. Studies analyzing activities involving jumps and the evaluation of force by muscle torque did not show any effects on bone geometry either.

## DISCUSSION

Most studies used DXA evaluation method and the HSA software, followed by those using pQCT for quantitative evaluation of BMD, BMC, and bone geometry. Use of the MRI was less frequent, as only one article with this method was included in this review. We analyzed studies conducted with children and adolescents up to 18 years of age, an important phase for development and bone growth peak, and it led us to state that the practice of physical activity and/or sports offers benefits to the evaluated bone parameters.

All studies included in the review and addressing the practice of gymnastics presented higher values of DXA parameters, such as whole-body BMD and BMC, bone geometry assessed by pQCT, including femur and intertrochanteric area BMD volume, compared to individuals of the same age who did not practice any kind of activity. This difference has been consolidated in the literature, since gymnastics athletes present increased BMD when compared to non-athlete girls of the same age, and this can be attributed to the impact forces imposed by jumping and falling actions in this sport.^38^

Practitioners of other sports, such as soccer, tennis and *capoeira*, had better values of BMD and bone geometry than control subjects and, in the case of soccer players, also compared to swimmers, which shows that sports requiring impact and body overload promote bone deposition, thus helping to improve peak bone density.

Three articles comparing swimmers with practitioners of other sports to controls were found. In the cross-sectional study by Ferry et al.,^30^ while female soccer players had higher BMD values and bone geometry parameters compared to controls, swimmers presented lower values than the control group for several parameters, including as BMD and cross-sectional area, even though they presented increased Buckling Ratio (BR) Z score, which is the ratio of outer ray and bone wall thickness. In other words, BR is the deformation rate estimated in the HSA by modeling the ring’s circular or elliptical cross-section with a fixed ratio (60, 70 and 100%) from the cross-sectional area (CSA) in the cortical shell to femoral neck regions), from intertrochanteric (IT) and femoral axis (FA) regions, respectively.^30^

In a longitudinal study by the same author,^37^ swimmers were reported to have increased values in some areas, such as IT area’s BMD, cross-zone Z score and BR. However, these swimmers would train more frequently per week (10h/week), swimming ±5.7 km per session, on average, which suggests that high frequency and intensity of activities may contribute to such result. These findings are in agreement with a systematic review that found most studies reporting similar bone density and geometry values between swimmers and control subjects, meaning that swimming is not sufficient to stimulate bone growth above regular standards,^11^ and the intensity of trainings should be increased so that the stimulus goes beyond this standard.

Other forms of physical activity assessment, such as questionnaires, provide strong evidence that the more intense and frequent the physical activities, the better the results in bone parameters. Michalopoulou et al.^24^ analyzed individuals classified as practitioners of high and low-intensity physical activities, the former group showing better bone geometry results. Alwis et al.^8^ noted that males who dedicated more time to physical education classes obtained better results for the third lumbar vertebra compared to males with less time.

In a cross-sectional study using physical evaluation and jumps, the influence of physical activity was proven significantly correlated with the following bone parameters: total bone area, total bone density, bone strength index, cortical area, and bone deformation index in males and bone strength index in females, whereas vertical jumps were correlated only with bone strength index for females, indicating low influence in a group of individuals who did not practice regular physical activity or sports.^23^ When it came to isolated jumping exercises at two different heights, no significant difference between the groups before and after the exercise programs were found, regardless of the height of the jumps.^32^

Nevertheless, in our review we found more cross-sectional than longitudinal studies (eight studies, representing 38% of the sample) (Table 2). Among these, two studies^9^ ^,^ ^32^ had no differences in bone geometry parameters evaluated in individuals after a period of intervention with physical education classes or jumping exercises, unlike other longitudinal studies, in which gymnasts,^33^ football^37^ and capoeira practitioners^35^ had better results in bone geometry parameters compared to sedentary subjects after a period of intervention. In the study by Ducher et al.^36^, significant differences were found between the arm used to play and the other arm of tennis players, with increase in bone geometry values for the most used one.

It is important to highlight some limitations of these studies. For example, the additional time of physical education classes was not enough for the group that also participated in classes for a shorter time,^8^ ^,^ ^9^ or even the absence of a control group and the use of the dominant limb for comparison in individuals who performed jumping exercises,^32^ being disregarded the fact that increase in bone mass also occurs through osteometabolic action in bone tissue as a whole, not only in isolated regions. These limitations raise doubts as to the practice of exercises and physical activities, adding bias to the analyzes and making assertive conclusions impossible. In addition, only one article addressing the practice of *capoeira* and one with tennis players were found in our research and, therefore, the information about these sports is insufficient to draw any conclusion.

It is worth mentioning, though, that these findings may be related to the time and frequency of activities practiced. For example, the results of the study with gymnasts who would practice from 6 to 16h/week were superior when compared to those of gymnasts practicing 1 to 5h/week; this can lead to adaptations related to the bone dynamic structure, which is remodeled according to the external forces it is subjected to. It all means that the ability of the bone to self-organize in size, shape and structure depends on the mechanical loads it is subjected to (Wolff’s Law). Frost & Schonau^39^ proposed that the development of bone resistance depends on muscle action, as the muscles generate the greatest pressure and mechanical load on the bones. Therefore, sports demanding muscle tension above the necessary threshold will promote more bone resistance than sports with submaximal tension.^38^

Finally, most studies used gymnastics as the sport to be analyzed, and the number of studies on collective sports (soccer only) was minimal, with no other modalities practiced by children and adolescents. Thus, there is a shortage of studies and the need for further research on sports and physical activities that can greatly influence bone geometry of children and adolescents, so one can demonstrate the effects of many modalities that have been little studied in this age group.

In conclusion, all studies of this review showed gymnastics as having positive influence on bone geometry, as well as soccer and more intense exercises measured by questionnaire. Therefore, such specific, more frequent and intense activities are suggested to positively affect bone geometry parameters.
